# Readministration of Cancer Drugs in a Patient with Chemorefractory Metastatic Colorectal Cancer

**DOI:** 10.1155/2020/2351810

**Published:** 2020-06-23

**Authors:** Tetsuro Kawagoe, Go Ikeda, Yu Oshiro, Yuta Maruki, Keiko Kaneko, Katsuhiko Iwakiri

**Affiliations:** Division of Gastroenterology, Department of Internal Medicine, Nippon Medical School, Japan

## Abstract

A 63-year-old woman was admitted to our institution for severe pain in her right lower abdomen caused by the perforation of cecal cancer. She underwent emergency surgery, from which she was diagnosed with cecal carcinoma with liver, lung, and lymph node metastases. As she was taking aspirin to prevent cerebral infarction, anti-vascular endothelial growth factor (receptor) antibody and regorafenib therapy were not used. Thus, we started a modified FOLFOX 6+cetuximab regimen. This first-line treatment initially achieved a partial response (PR), but she then developed progressive disease (PD) after 14 months. We changed the regimen to FOLFIRI, followed by trifluridine/tipiracil, but her progression-free survival periods were 2.7 months and 1 month, respectively. Although we cycled through the available array of standard cancer drugs, the patient showed a good performance status, and some benefit from treatment still seemed plausible. We readministered the 5-fluorouracil oral preparation S-1, which maintained stable disease (SD) for 7 months. After PD emerged, we readministered the anti-epidermal growth factor receptor (EGFR) antibody panitumumab for 7.5 months of SD. Finally, 39 months after her diagnosis, she died from rapidly progressing disease. However, her relatively long survival implies that readministering drugs similar to those used in previous regimens might benefit patients with metastatic colorectal cancer.

## 1. Introduction

Chemotherapy is usually the first-choice treatment for metastatic colorectal cancer (mCRC). Relatively new cytotoxic agents (such as irinotecan and oxaliplatin) and molecular targeted agents (such as antibodies against vascular endothelial growth factor (receptor) (VEGF (R)) and epidermal growth factor receptor (EGFR)) have extended the median overall survival of patients with mCRC to more than 20 months [1–3]. Guidelines recommend chemotherapy regimens with standard cancer drugs, such as 5-fluorouracil (FU), irinotecan, oxaliplatin, anti-VEGF (R) or anti-EGFR antibodies, regorafenib, and trifluridine/tipiracil [4–6]. More recently, immunotherapy has become available for a minority of patients with microsatellite instability-high tumors [7]. Although patients who have cycled through these standard chemotherapies are usually only treated palliatively, some selected patients can maintain good performance status (PS) and receive further chemotherapies. For such patients, there are limited further chemotherapeutic options. Here, we report a woman with mCRC who benefitted from the reintroduction of S-1, an oral prodrug of 5-FU, and anti-EGFR antibodies after receiving standard 1^st^-, 2^nd^-, and 3^rd^-line chemotherapies.

## 2. Case Presentation

A 63-year-old woman with a medical history of hypertension and cerebral infarction was admitted to our hospital with severe abdominal pain in October 2012.

**Figure 1:**
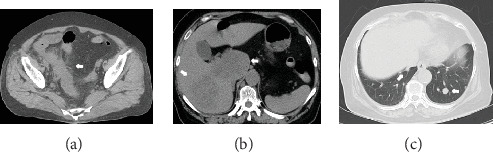
Pelvic CT showed inflammation spread and abscess formation around the cecum (a). Abdominal CT showed a huge mass and multiple nodules in the liver (b). Chest CT showed nodules in the lungs (c). Arrows: lesions.

**Figure 2:**
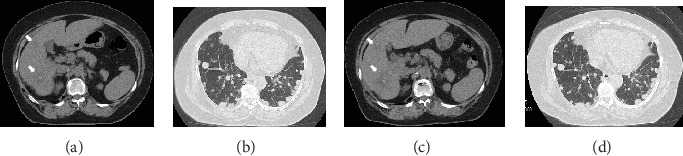
Abdominal and chest CT before the administration of S-1 (a, b). Stable disease after therapy (c, d). Arrows: lesions in the liver.

**Figure 3:**
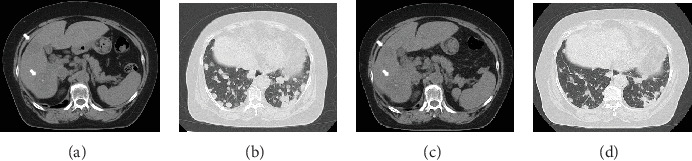
Abdominal and chest CT before the administration of panitumumab (a, b). Stable disease after therapy (c, d). Pulmonary metastasis was slightly reduced after panitumumab treatment. Arrows: lesions in the liver.

Computed tomography (CT) scan of the abdomen and pelvis showed inflammation spread, abscess formation, lymphadenopathy around the cecum, and a huge mass with multiple nodules in the liver (Figures 1(a) and 1(b)). A chest CT also revealed multiple pulmonary nodules (Figure 1(c)). She was clinically diagnosed with intestinal perforation owing to cecal cancer and underwent emergency surgery. She was intraoperatively diagnosed with obstruction of the appendicular root owing to cecal cancer, perforation of the vermiform appendix, intraperitoneal abscess, and lymphadenopathy around the cecum and received an ileocecal resection, D1 lymph node dissection, and a peritoneal wash. After surgery, she was finally diagnosed with moderately differentiated wild-type *KRAS* adenocarcinoma of the cecum (stage: T3N1M1b, per the Union for International Cancer Control criteria). A microsatellite instability (MSI) test was not performed. *RAS* and *BRAF* status were also not investigated. We initiated therapy using cetuximab (500 mg/m^2^; 14-day cycle) and the mFOLFOX6 regimen (5-FU 400 mg/m^2^ bolus injection; leucovorin (LV) 200 mg/m^2^, 46 h continuous infusion with 5-FU 2400 mg/m^2^; and oxaliplatin 85 mg/m^2^; 14-day cycle) in October 2012. This treatment resulted in 7.75 months of partial response (PR), followed by a stable disease (SD) period of 6.25 months and progressive disease (PD) for a total progression-free survival (PFS) period of 14 months. As a 2^nd^-line treatment, we started the FOLFIRI regimen (5-FU 400 mg/m^2^ bolus injection, LV 200 mg/m^2^, 46 h continuous infusion with 5-FU 2400 mg/m^2^, and irinotecan 150 mg/m^2^; 14-day cycle), but she developed PD after 2.7 months. We started trifluridine/tipiracil (35 mg/m^2^ administered twice daily on Days 1–5 and Days 8–12 of a 28-day cycle) as a 3^rd^-line treatment, but this led to PD after 1 month. As this patient had a history of cerebral infarction and used antiplatelet drugs, anti-VEGF (R) antibody and regorafenib therapies were contraindicated. Hence, at this stage, no new standard cancer drugs could be tried. However, the patient's general condition was still good, and she requested further chemotherapy. Therefore, we readministered the 5-FU oral preparation S-1 (80 mg/m^2^, Days 1–28, 42-day cycle), which provided a 7-month SD period (Figure 2). When PD was again confirmed, we administered panitumumab (6 mg/kg once every 2 weeks) as an anti-EGFR antibody rechallenge. The patient achieved SD on this regimen for 7.5 months (Figure 3). Finally, 39 months after her diagnosis, the patient died because of rapid disease progression. While receiving readministered drugs, her PS was well maintained; she suffered no grade ≥ 3 adverse events (per the National Cancer Institute Common Toxicity Criteria, version 4.0; Table 1).

## 3. Discussion

Although this was a case of unresectable CRC, its treatment can be considered successful because the patient survived for 39 months after diagnosis while maintaining good PS despite being unable to receive anti-VEGF (R) antibody therapy. Because she survived for 14.5 months after cycling through standard cancer regimens, retreatment apparently facilitated her relatively long survival.

Several reports on readministering anticancer drugs [8–16] have suggested that after a washout period, earlier-line drugs that were initially effective but then became ineffective might become effective again [11–15]. Santini et al. reported cetuximab rechallenge to be significantly effective after a cetuximab-free interval with cytotoxic chemotherapy [13]. Their hypothesis is as follows. If the anti-EGFR antibody was initially effective, it would have reduced EGFR-sensitive clonal cells and insensitive clones predominate at the PD stage. Subsequent cytotoxic chemotherapy would reduce the number of insensitive clones, and at the time of PD, sensitive clones would grow and become dominant again. As a result, the anti-EGFR antibody would become active again. Their hypothesis appears to be supported by the result of the CRICKET trial [14], in which rechallenge treatment with cetuximab was demonstrated to be effective, especially in patients without *RAS* mutations in circulating tumor DNA. This hypothesis implies that even if the readministered drug is panitumumab rather than cetuximab, an effect can be expected because both drugs are anti-EGFR antibodies. Panitumumab might also have been effective after cetuximab because panitumumab is a fully human monoclonal antibody (MoAb), whereas cetuximab is a chimeric MoAb consisting of ~30% mouse protein and may have a different sensitivity to cancer. Few reports have investigated the effect of panitumumab after progression on cetuximab in colorectal cancer patients. Marino et al. reported in their retrospective study that treatment with panitumumab after progression on cetuximab was effective because its PR and SD rates were 5% and 25% in 20 patients, respectively, and the median PFS and OS were 5 and 8 months, respectively [15]. In contrast, Wadlow et al. reported no responders to panitumumab treatment after progression on cetuximab and a SD rate of 45% with a median duration of only 1.7 months in their single-arm phase II trial of 20 patients [16]. Because both of these trials were conducted in a small number of patients, and panitumumab treatment after progression on cetuximab was actually effective in our study, it is necessary to identify the type of cases in which panitumumab treatment after progression on cetuximab is effective in the future. S-1 was developed to improve the therapeutic effect of tegafur, an oral fluoropyrimidine, by maintaining high 5-FU concentrations in plasma and tumors with less gastrointestinal toxicity by 5-chloro-4-dihydroxypyridine (CDHP) and potassium oxonate. In this case, the mechanism by which S-1 (as an oral fluoropyrimidine agent) was effective after the 5-FU infusion regimen became ineffective is unclear. Reportedly, the use of the FOLFOX regimen with 5-FU infusion enhances dihydropyrimidine dehydrogenase (DPD) activity in tumors [17]. In the present case, S-1 may have maintained high levels of 5-FU in the tumor by suppressing DPD activity enhanced by pretreatment with CDHP.

## 4. Conclusion

In conclusion, we report a patient with metastatic CRC for whom repeated standard cancer treatments were effective despite prior development of refractory reactions. Why retreatment was effective remains unclear. Further research is needed.

## Figures and Tables

**Table 1:** Treatment toxicities and performance status.

	mFOLFOX6+Cetu	FOLFIRI	Trifluridine/tipiracil	S-1	Panitumumab
Grade 1/2					
Decreased WBCs	○	○			
Decreased neutrophils		○	○		
Anemia	○	○	○	○	○
Decreased platelets	○			○	
Fever	○		○	○	○
Anorexia	○		○	○	
Dry skin	○				○
Peripheral neuropathy	○	○			
Insomnia	○				
Paronychia	○				○
Fatigue		○	○	○	
Mucositis oral				○	○
Diarrhea				○	
Rash acneiform					○
Grade 3					
Decreased neutrophils	○				
PS	1	1	2	1	1

Cetu: cetuximab; WBC: white blood cells.

## Data Availability

The clinical data used to support the findings of this study are included within the article.
